# Different body components associate differently with diet quality and physical activity in men and in women: a cross-sectional analysis

**DOI:** 10.7717/peerj.21821

**Published:** 2026-10-21

**Authors:** Nima Hosseini-Akhavan, Bahri Bektashi, Adrian V. Jaeggi, Nicole Bender

**Affiliations:** Institute of Evolutionary Medicine, University of Zurich, Zurich, Switzerland

**Keywords:** Body composition, BMI, Obesity, Diet composition, Nutrition, Lifestyle, Evolution of body composition, Sex differences

## Abstract

**Background/objectives:**

While the association between food quantity and obesity is well studied, the impact of diet components on specific body compartments is less well understood. Furthermore, there seem to be sex differences in the metabolism of different foods and the response towards physical activity (PA).

**Methods:**

We investigated the association between diet components using a food frequency questionnaire, and body composition using bioimpedance analysis. We assessed data from 2021 of a cohort study of the Swiss general population, including 435 persons of both sexes (age range of 19–84 years). We used multivariable linear regression models separated by sex to assess the association between body composition (body mass index (BMI), skeletal muscle mass index (SMI), fat mass index (FMI), and visceral fat (VAT)), and diet quality and PA. Age, education, and place of residence were included as potential confounders.

**Results:**

For both sexes, age showed the strongest positive association with FMI and VAT, while PA was negatively associated, especially in men. In men PA was also associated with BMI (not in women). Diet quality showed associations with body composition measures in women (not in men). In women, meat consumption was positively associated with SMI and FMI (not in men). In men fruits and wholegrain foods were negatively associated with FMI. VAT was negatively associated with fruits in women, and with fruits and wholegrain foods in men.

**Conclusion:**

Our results are in line with evolutionary hypotheses on sex specific energy allocation and might contribute to more precise diet recommendations.

## Introduction

According to the WHO there was a worldwide rapid increase of obesity in the last 30 years. In 2014 over 1.9 billion adults lived with overweight and 600 million lived with obesity (WHO, 2016). Adiposity was associated with the metabolic syndrome, diabetes type 2 (T2D), cardiovascular disease (CVD), certain cancers, and higher overall mortality (Lee et al., 2018; Mili et al., 2021). However, disease risk varies with the type of fat tissue, as each large fat deposit shows unique characteristics (Abate et al., 1995; Kelley et al., 2000). Fat storage can be divided into lower and upper body fat. Lower body fat is considered all adipose tissue caudal to the inguinal area. Upper body fat is stored in the upper extremity, breast in women, and mainly in the abdominal area (Jensen, 2020). Abdominal fat can be divided into three areas: subcutaneous fat, retroperitoneal fat, and visceral fat (VAT), which surrounds vital organs (Prasad et al., 2011).

Studies in the 1970s and 1980s showed a correlation between obesity and high blood pressure, hypertriglyceridemia, hyperuricemia, and glucose intolerance that can lead to T2D and the metabolic syndrome (Reaven, 1997; Phillips, 1978). Later studies considered upper body fat, stored as abdominal fat (so-called central obesity), whether subcutaneous or visceral, associated with an increased risk of T2D and CVD (Prasad et al., 2011; Kelley et al., 2000). Especially visceral and deep subcutaneous fat was found to be associated with insulin resistance and the metabolic syndrome (Kelley et al., 2000; Abate et al., 1995).

Furthermore, fat deposits can be considered as biochemically active tissues. If abundant, they can create an inflammatory environment with increased levels of C-reactive protein (CRP), fibrinogen, plasminogen activator-inhibitor-1 (PAI-1), cytokines, adhesion molecules, and the secretion of further adipokines (adiponectin, resistin, leptin, tumor necrosis factor-α, and interleukin-6) (Haffner, 2007). This inflammatory activity and the released adipokines can increase the risk for atherosclerosis and CVD due to thrombosis at the vessel endothelium (Landsberg et al., 2013).

Moreover, the specific location of VAT as biochemically active fat tissue surrounding vital organs and central vessel systems can lead to a higher release of free fatty acids (FFA) and inflammatory factors, and eventually to a higher mortality due to increased atherosclerosis, T2D, and cardioembolic disease (Neeland et al., 2019). There is also an association between VAT and a higher release of FFA and inflammatory factors into the portal vein and hepatic system, as this is the primary drainage system for VAT (Fontana et al., 2007).

Despite the health disadvantages of obesity and abundant VAT, the human body shows the tendency to accumulate large amount of fat tissue in an obesogenic environment. Known proximate risk factors of obesity are among others age, sex, ethnicity, diet, and physical activity (PA) (Maskarinec et al., 2017). Neel (1962) argued that the capacity to accumulate large amounts of fat ensured a survival advantage in human evolution, shaping our genome. According to this ‘thrifty gene’ hypothesis, accumulating fat has been a survival strategy to overcome periods of food shortage (Neel, 1962). In our modern society, these genetic variants are now disadvantageous since there are no longer episodes of food scarcity in developed countries (Speakman, 2008; Neel, 1962). Due to technological development, less PA is required, and many occupations today are performed in a sedentary position. The abundance of highly processed and calorie-rich food, and lack of PA has led to excessive and unused fat storage (Chan & Woo, 2010).

The different patterns for accumulating fat tissue and strategies for using energy in modern humans was found to show a wide variety according to age, sex, ethnicity or social status (Wells, 2012). For instance, there seems to be a sex-specific difference in the metabolism of different foods. Men and women metabolize food differently and respond differently to PA, mainly due to genetic and hormonal differences (Pucci et al., 2017). Ultimate, evolutionary explanations discuss the sex difference in fat accumulation patterns as specific adaptations in female energy storages for pregnancy and lactation (Vernon & Pond, 1997), especially in the view of the large and energy-demanding brain of the offspring (Crawford, 2012). The pregnancy period is also regarded as relevant for epigenetic priming of the offspring, leading to a higher propensity to accumulate fat later in life if the mother experienced negative environmental conditions during pregnancy such as under- or malnutrition. These ideas were formulated as the *Thrifty Phenotype Hypothesis* and the *Developmental Origins of Health and Disease* (DOHaD) hypothesis (Gluckman, Hanson & Low, 2019).

Today, diet and PA are among the few factors that we can influence individually to achieve better health outcomes. While the association between food quantity and obesity was studied extensively, the impact of specific diet components on specific fat tissues is less well understood. In fact, not all foods seem to have the same effect on weight gain and fat storage in men and in women (Panizza et al., 2020; Van Eekelen et al., 2019).

The present study therefore aimed at gaining insight into the association of diet and PA with body composition (fat mass index (FMI), VAT, skeletal muscle mass index (SMI), and body mass index (BMI)), focusing on sex differences and on the relative importance of specific food types.

## Methods

### Study procedures

The participants of this study were recruited from an ongoing cohort study that started on September 23, 2019, in Zurich, Switzerland. The participants were initially recruited from a previous cohort, and from 2021 onwards additionally *via* invitations, mailing lists, and advertisements. Inclusion criteria were a minimum age of 18 years and German reading skills to be able to answer the questionnaire. Exclusion criteria were pregnancy and implanted electrical devices. The study procedure was explained to the participants both verbally and in writing. Informed consent was signed by the participants and by the researchers before data collection. The data was collected at the University of Zurich with a body composition analyser (bioimpedance analysis, BIA) and with a questionnaire containing quality and quantity of food intake, the PA level at work and during leisure, and sociodemographic factors such as age, sex, education level, and place of residence. Here we analyse the data collected in 2021, as this was the year with the most participants. This study has been performed in accordance with the ethical standards laid down in the 1964 Declaration of Helsinki and its later amendments. The study was approved by the Ethics Committee of Kanton Zurich (BASEC-Nr. 2020-00654).

We used a food frequency questionnaire that was previously developed and validated by ETH Zurich, Switzerland (Hagmann, Siegrist & Hartmann, 2019). The questionnaire was an adapted version based on the food frequency questionnaire of the Nurses’ Health Study (Hu et al., 2016). It allowed for the assessment of a diet quality index, according to the participants’ adherence to official Swiss dietary recommendations in five food categories (Hagmann, Siegrist & Hartmann, 2019). The food portion sizes were adapted to the official Swiss dietary recommendations (BLV, 2017).

All measurements were carried out at the ScanLab of the University of Zurich, Switzerland, by trained personnel. We analysed body composition with a segmental medical 8-point body composition bioimpedance analyser (BIA) (Seca mBCA 515, Seca AG, Reinach, Switzerland) (Peine et al., 2013). This device uses 8 electrodes (4 on hands and 4 on feet) to send weak, painless currents through the body. It calculates segmental body composition—muscle, fat, and water—by measuring resistance across arms, legs, and torso. Using validated algorithms, it calculates whole body fat mass, visceral fat mass, skeletal muscle mass (total body and all for limbs separately), and whole-body water/extracellular water. This device was validated before in a multi-ethnic population (Day et al., 2018). The measurements must be taken barefoot with both hands and feet on the four foot and four hand electrodes of the device. Weight and height were measured with an incorporated scale and a standard stadiometer (Seca 274). To calculate VAT, waist circumference (WC) must be measured manually and entered into the BIA software. WC was measured by trained personnel using a hand-held stretch-resistant tape with automatic retraction (Seca 201) and according to WHO standards (WHO, 2008). These standards foresee the measurements at the midpoint between the pelvis’s highest point and the ribcage’s lowest point. The Seca BIA software determined BMI, FMI, fat-free mass (FFM), VAT, SMI, total body water (TBW), and extracellular water (ECW). All measurements were carried out with the same devices during the whole study.

As there was variation in the time of day when the BIA measurements were taken, the exact time of the measurement was recorded. Participants were informed in advance not to eat, drink, and exercise at least two hours before measurement. Additionally, participants had to indicate their last time of eating, drinking, and PA in the questionnaire.

### Variables

The questionnaire contained questions about sociodemographic characteristics such as age, sex, education, and place of residence. As age showed a bimodal distribution, age was categorized in four quartiles with 1 as the youngest quartile and 4 as the oldest quartile. Education was recorded in the following categories relevant to the Swiss educational system: (1) mandatory education, (2) basic education, (3) professional training, (4) high school, (5) higher professional studies, (6) higher education, (7) University, and (8) None. For sample size reasons (see Table 1 for frequencies within the original categories) we regrouped the data into three groups for statistical analyses: basic education (1–3 and 8), higher education (4–5), and tertiary education (6–7). Place of residence was recorded as (1) urban, (2) suburban, and (3) rural.

**Table 1:** Descriptive variables of the sample.

**Variable**	**Men**	**Women**	**Difference**
Sample size (n, %)	190 (43.7)	245 (56.3)	*p* < 0.01
Age (years) (mean, SD)	41.7 (18.4)	36.1 (15.7)	*p* < 0.01
Education (n, %)			*p* = 0.130
Mandatory	1 (0.5)	1 (0.4)	
Basic	0 (0.0)	0 (0.0)	
Professional training	17 (8.9)	18 (7.3)	
High School	28 (14.7)	40 (16.3)	
Higher professional studies	19 (10)	21 (8.6)	
Higher education	26 (13.7)	12 (4.9)	
University	97 (51.1)	148 (60.4)	
None	0 (0.0)	1 (0.4)	
Missing	2 (1.1)	4 (1.6)	
Residence (n, %)			*P* = 0.024
Urban	100 (52.9)	150 (62.8)	
Agglomeration	56 (29.6)	67 (28.0)	
Rural	33 (17.5)	22 (9.2)	
Diet quality index (n, %)			*p* < 0.001
Score 0	8 (4.2)	9 (3.7)	
Score 1	81 (42.6)	48 (19.6)	
Score 2	50 (26.3)	77 (31.4)	
Score 3	35 (18.4)	57 (23.3)	
Score 4	14 (7.4)	44 (18.0)	
Score 5	2 (1.1)	10 (4.1)	
PA leisure (n, %)			*P* < 0.001
Very light	3 (1.6)	3 (1.2)	
Light	9 (4.7)	21 (8.6)	
Moderate	43 (22.6)	99 (40.4)	
Heavy	102 (53.7)	103 (42.0)	
Very heavy	31 (16.3)	17 (6.9)	
Missing	2 (1.1)	2 (0.8)	
PA work (n, %)			*P* = 0.124
Very light	125 (65.8)	142 (58.0)	
Light	42 (22.1)	76 (31.0)	
Moderate	16 (8.4)	23 (9.4)	
Heavy	3 (1.6)	1 (0.4)	
Missing	4 (2.1)	3 (1.2)	
Height (m) (mean, SD)	1.79 (0.1)	1.67 (0.1)	*p* < 0.001
BMI (Kg/m^2^) (n, %)			*p* < 0.001
<18.5	4 (2.1)	16 (6.6)	
18.5–24.9	120 (63.2)	197 (80.7)	
25–29.9	52 (27.4)	24 (9.8)	
≥30	14 (7.4)	7 (2.9)	
BMI (Kg/m^2^) (mean, SD)	24.4 (3.2)	22.2 (3.2)	*P* < 0.001
SMI (Kg/m^2^) (mean, SD)	9.4 (1.0)	7.0 (0.8)	*p* < 0.001
FMI (Kg/m^2^) (mean, SD)	2.1 (0.5)	2.5 (0.5)	*p* < 0.001
VAT (l) (mean, SD)	1.4 (1.2)	0.5 (0.6)	*p* < 0.001
TBW (l) (mean, SD)	45.6 (5.1)	32.2 (3.5)	*p* < 0.001
ECW (l) (mean, SD)	18.7 (2.1)	14.1 (1.6)	*p* < 0.001

**Notes.**

The difference between sample sizes was assessed using a one-sample binomial test. Differences in means were assessed using *t*-test and Wilcoxon-Mann–Whitney test. Differences between categorical variables were assessed using the Kruskal–Wallis equality-of-populations rank test and the chi-square test. PA, physical activity; BMI, body mass index; SMI, skeletal muscle mass index; FMI, fat mass index; VAT, visceral adipose tissue; TBW, total body water, ECW, extracellular water.

As mentioned, we used a food frequency questionnaire that was previously developed and validated by the Swiss Food Panel study (Hagmann, Siegrist & Hartmann, 2019). From this questionnaire, it is possible to calculate a diet quality index using five food categories: fruits, vegetables, wholegrain foods, meat and meat products, and sweet/salty snacks and alcohol. For each category, the official Swiss recommendations for minimum or maximum weekly intake are used as cut-off values. If a participant met the recommendation in a category, a point was assigned. In this way each participant obtained a score from 0 to 5 that reflected the overall healthiness of his/her diet (Hagmann, Siegrist & Hartmann, 2019). During our analyses we summarized the score into three larger categories because of sample size reasons (see Table 1 for original categories and for frequencies within the original categories): unhealthy (0–1), mediocre (2–3), and healthy (4–5). We assessed the last time of drinking in order to correct for liquid imbalances as follows: (1) less than one hour ago, (2) one hour ago, (3) two hours ago, and (4) three or more hours ago.

We further recorded PA during leisure time following a validated questionnaire (Johansson & Westerterp, 2008). For PA during leisure, the questionnaire asks the mean activity level performed during a typical year. The answers can be given choosing one of the following categories: (1) very light (almost no PA), (2) light (*e.g.*, walking, slow biking), (3) moderate (*e.g.*, running, biking), (4) heavy (*e.g.*, intensive running, intensive biking), and (5) very heavy (exhaustive activity several times per week) (Johansson & Westerterp, 2008). For analyses we divided these categories into three groups because of sample size reasons (see Table 1 for frequencies within the original categories), as light (1–2), medium (3), and heavy (4–5).

Physical activity at work was assessed with the same questionnaire (Johansson & Westerterp, 2008), offering the following categories to indicate the typical mean PA level at work: (1) very light (sitting at the computer or the desk for most part of the day), (2) light (work in sales, light manual work), (3) Moderate (work in a kitchen, cleaning, courier), and (4) heavy (heavy manual work, construction, agriculture). For the analyses, the categories 3 and 4 were fused (see Table 1 for frequencies within the original categories). For both types of PA, examples for each category were given in the questionnaire, to facilitate the correct choice of individual categories.

We calculated BMI from weight divided by the square of height (Kg/m^2^) and classified it according to the official WHO classification into underweight (<18.5 kg/m^2^), normal weight (18.5—<25 kg/m^2^), overweight (25—<30 kg/m^2^) and obesity (≥30 kg/m^2^) (WHO, 2008). Similarly, we calculated SMI as skeletal muscle mass divided by the square of height (Kg/m^2^), and FMI as fat mass divided by the square of height (Kg/m^2^). In this way we corrected for different body heights of the participants.

### Statistics

We compared the sample sizes of women and men using a one-sample binomial test, and the Wilcoxon-Mann–Whitney test for not normally distributed variables (age, BMI, SMI, FMI, VAT, TBW, ECW) to compare variables between the two sexes. Body height was normally distributed in both sexes and therefore compared with a two-sample *t*-test. Categorical data (residence) was assessed with the chi-square test, and differences between ordinal categorical variables (education, PA leisure, PA work, diet quality index, BMI categories) were assessed with the Kruskal–Wallis equality-of-populations rank test.

The association between body composition (BMI, SMI, FMI, and VAT) as dependent variables, and lifestyle variables (diet quality and PA) as independent variables were assessed by multivariable linear regression models, separately for each outcome. We tested sex as a potential effect modifier and found that in fact, sex showed to be an effect modifier of the association between SMI and diet/physical activity (*p*-values for interaction < 0.05), as well as of the association between VAT and diet/physical activity (*p*-values for interaction < 0.05). We therefore analysed our data separated by sex. We chose multivariable linear regression models to assess not only the main effects and how they influence each other, but also potential confounding effects by a multitude of variables, such as age, education, residence, and hydration status (*i.e.,* time since the last drink). In fact, age and socioeconomic variables are known to be potential confounders in epidemiological studies, and hydration status is relevant when assessing body composition using BIA (Schierbauer et al., 2023). We square root transformed not normally distributed variables before including them in the models (BMI, SMI, FMI, and VAT). The statistical software STATA version 14 was used (StataCorp, 2015).

## Results

### Descriptive data

In total, 435 persons (190 men and 245 women) participated in the study in the year 2021. The age range was 19-84 years. The characteristics of the participants by sex are given in Table 1.

There were more women than men in our sample (245 *vs* 190, *p* < 0.01). Women were younger than men (36.1 ± 15.7 *vs* 41.7 ± 18.4 years, *p* < 0.01), lived more often in cities (*p* = 0.024), but had a similar level of education than men (*p* = 0.130). Women ate healthier than men (*p* < 0.001), while men exercised more rigorously during leisure time (*p* < 0.001). Physical activity at work was similar between the sexes (*p* = 0.124). In terms of anthropometry, women were smaller, had a lower BMI, lower SMI and VAT, and less total and ECW than men (all *p* < 0.001). However, women had a higher FMI (*p* < 0.001). For all results see Table 1.

### Determinants of body composition

As sex showed to be an effect modifier of the association between SMI and diet/physical activity (*p*-values for interaction < 0.05), as well as of the association between VAT and diet/physical activity (*p*-values for interaction < 0.05), we show all analyses separated by sex. The multivariable linear regression models showed that in women, there was an association between a healthy diet and a low BMI (*p* = 0.02), low SMI (*p* = 0.015) and a low FMI (*p* = 0.038). PA during leisure was not associated with BMI (*p* > 0.05). However, PA was positively associated with SMI (*p* = 0.001), and at the same time negatively associated with FMI (*p* = 0.001) and VAT (*p* < 0.001). There was no association between PA at work and body composition in women.

In women, there were no direct associations with education and place of living with anthropometric measures in our sample (all *p* > 0.05). For women, BMI, FMI, and VAT were positively associated with age, while SMI was negatively associated with age (*p* < 0.05 for some of the middle age categories and all of the oldest age categories, compared to the youngest age categories). For more results see Figs. 1 and 2, and Supplemental Information 3.

**Figure 1:**
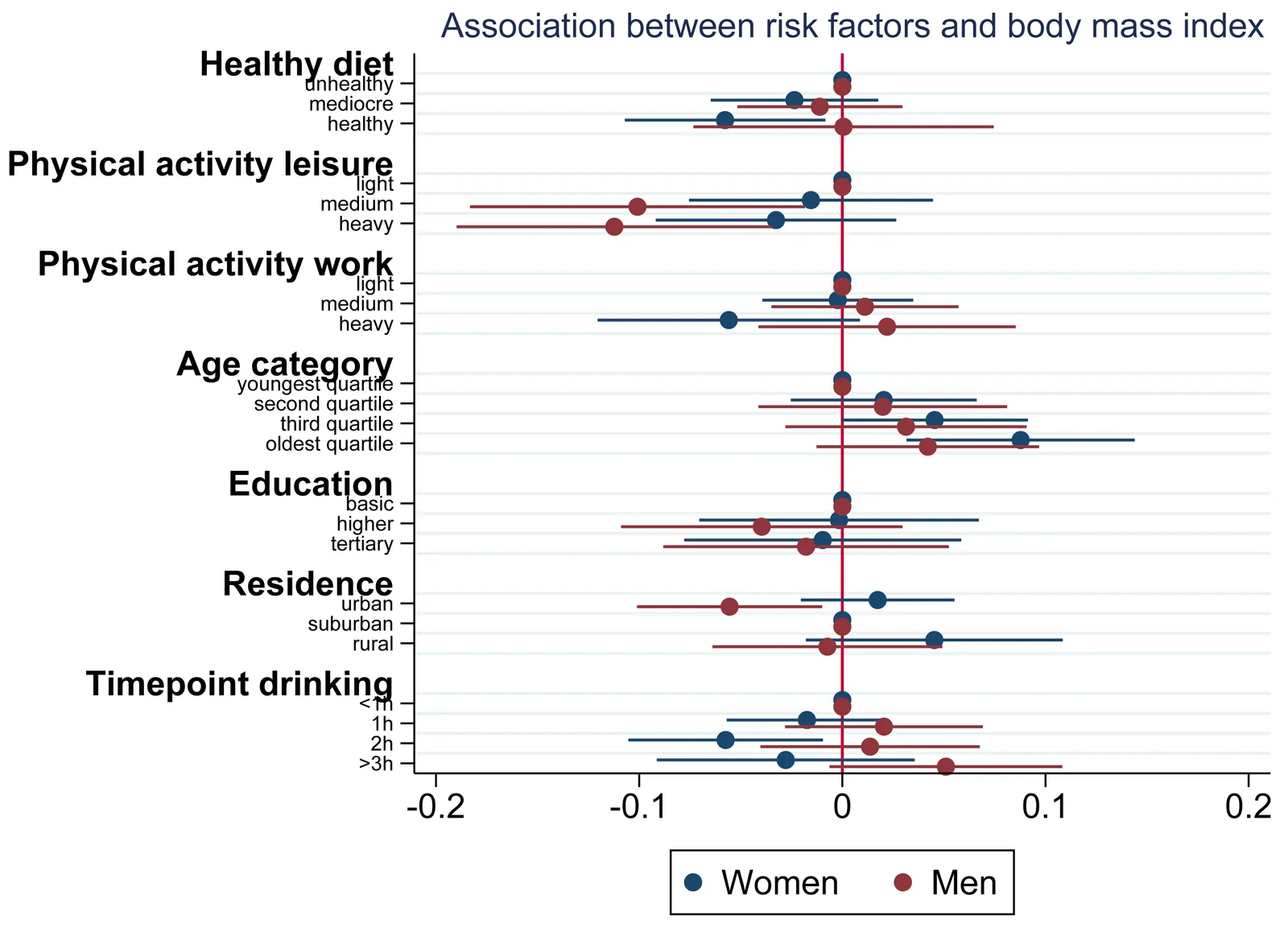
Plot of coefficients and 95% confidence intervals from multivariable linear regression analysis with outcome BMI, by sex. A coefficient of 0.09, as seen *e.g.*, for the oldest age category in women, implies an increase in BMI by 0.09 units per consumption on the square root scale compared to the youngest group.

**Figure 2:**
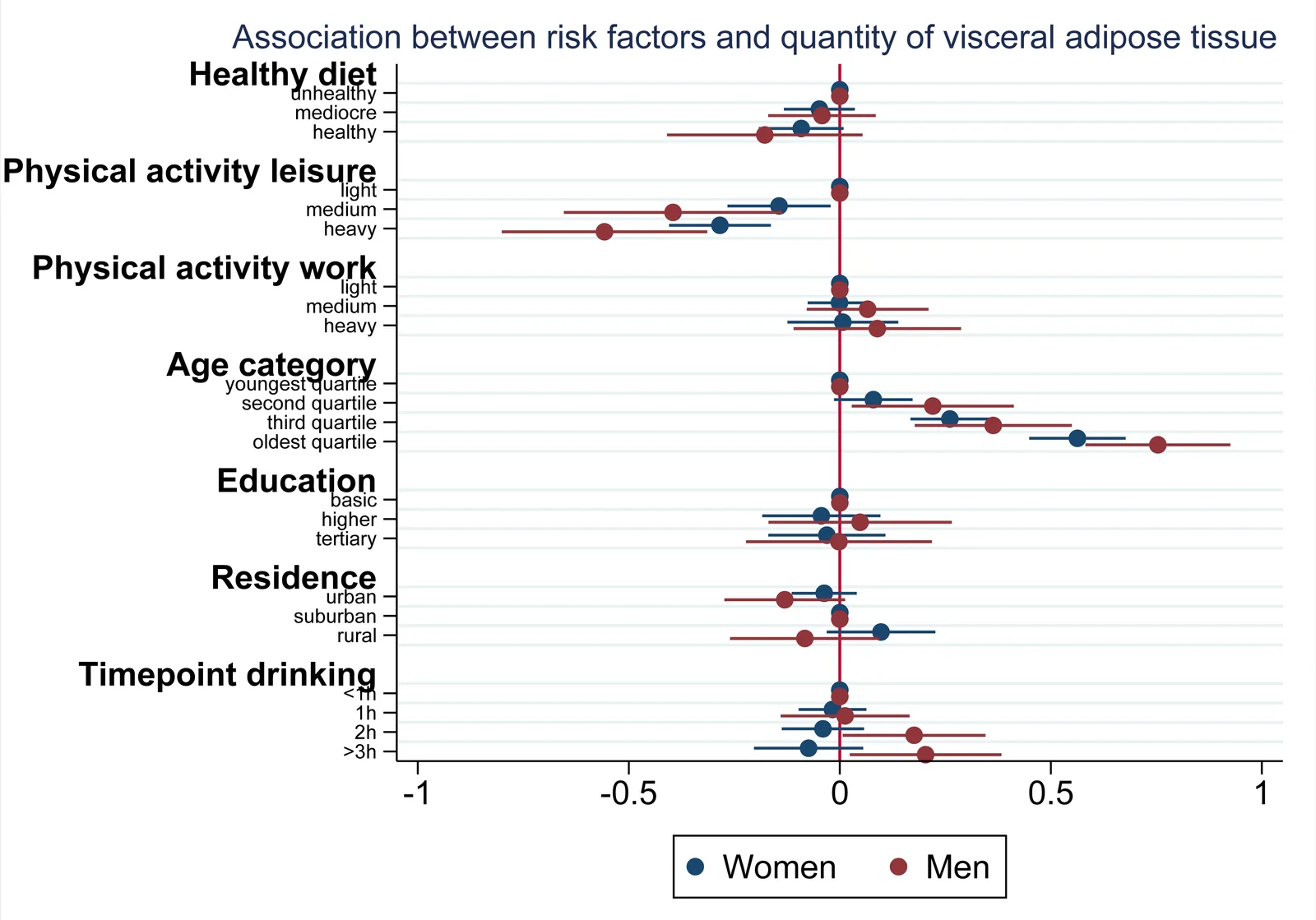
Plot of coefficients and 95% confidence intervals from multivariable linear regression analysis with outcome VAT, by sex. A coefficient of 0.56, as seen *e.g.*, for the oldest age category in women, implies an increase in VAT by 0.56 units per consumption on the square root scale compared to the youngest group.

In men, PA during leisure was negatively associated with BMI (*p* = 0.005), FMI (*p* < 0.001), and VAT (*p* < 0.001). Diet quality did not show significant associations to anthropometric measures in men (all *p* > 0.05). BMI (*p* = 0.017) and FMI (*p* = 0.031) were negatively associated with living in a city as compared to living in a suburban environment in men. There was no association of education with anthropometric measures in our sample for men (all *p* > 0.05). In men, the oldest category showed a negative association with SMI as compared to men in the youngest category. FMI and VAT were positively associated with age in men (all *p* < 0.05). For more results see Figs. 1 and 2, and Supplemental Information 3.

### Dietary components and body composition

*Muscle mass:* In women, meat consumption was positively associated with SMI (*p* < 0.001), while in men the association between meat consumption and SMI was borderline (*p* = 0.05). However, as we did not correct for multiple testing, we do not consider the result in men further. No other associations with dietary components were found with SMI in men (all *p* > 0.05). For all results see Fig. 3.

**Figure 3:**
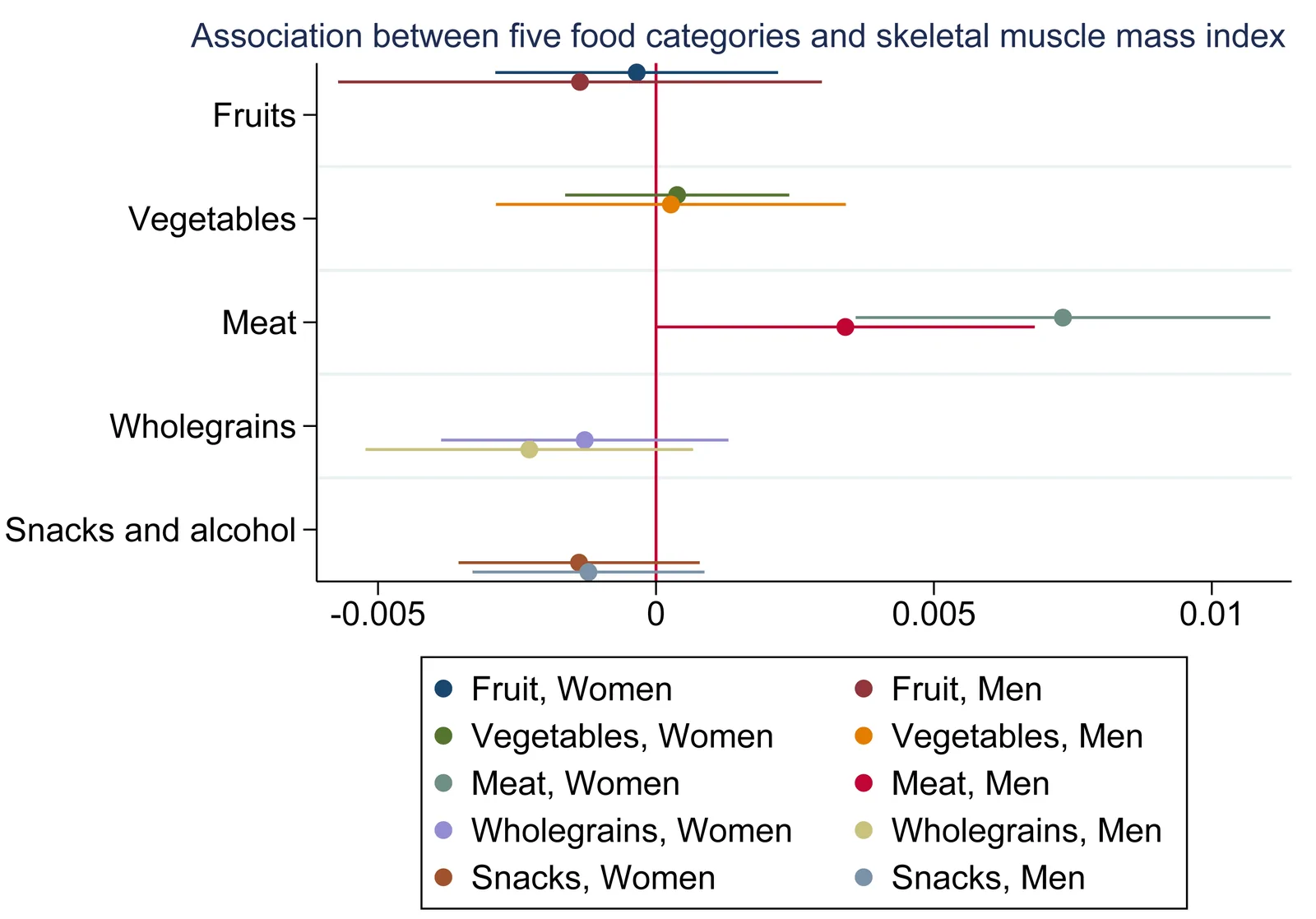
Plot of coefficients and 95% confidence intervals from several multivariable linear regression analyses with outcome SMI, by sex. Only the results for the dietary components (in number of consumptions per week) are shown. All analyses were conducted with the same covariables as in the models in Figs. 1 and 2. A coefficient of 0.007, as seen *e.g.*, for number of meat consumptions per week in women, implies an increase in SMI by 0.007 units per consumption on the square root scale.

*Fat mass:* In women, meat was positively associated with FMI (*p* = 0.006). In men, wholegrain foods (*p* = 0.001) were negatively associated with FMI. The consumption of sweet and salty snacks was not associated with FMI in neither sex in our sample (all *p* > 0.05). For all results see Fig. 4.

**Figure 4:**
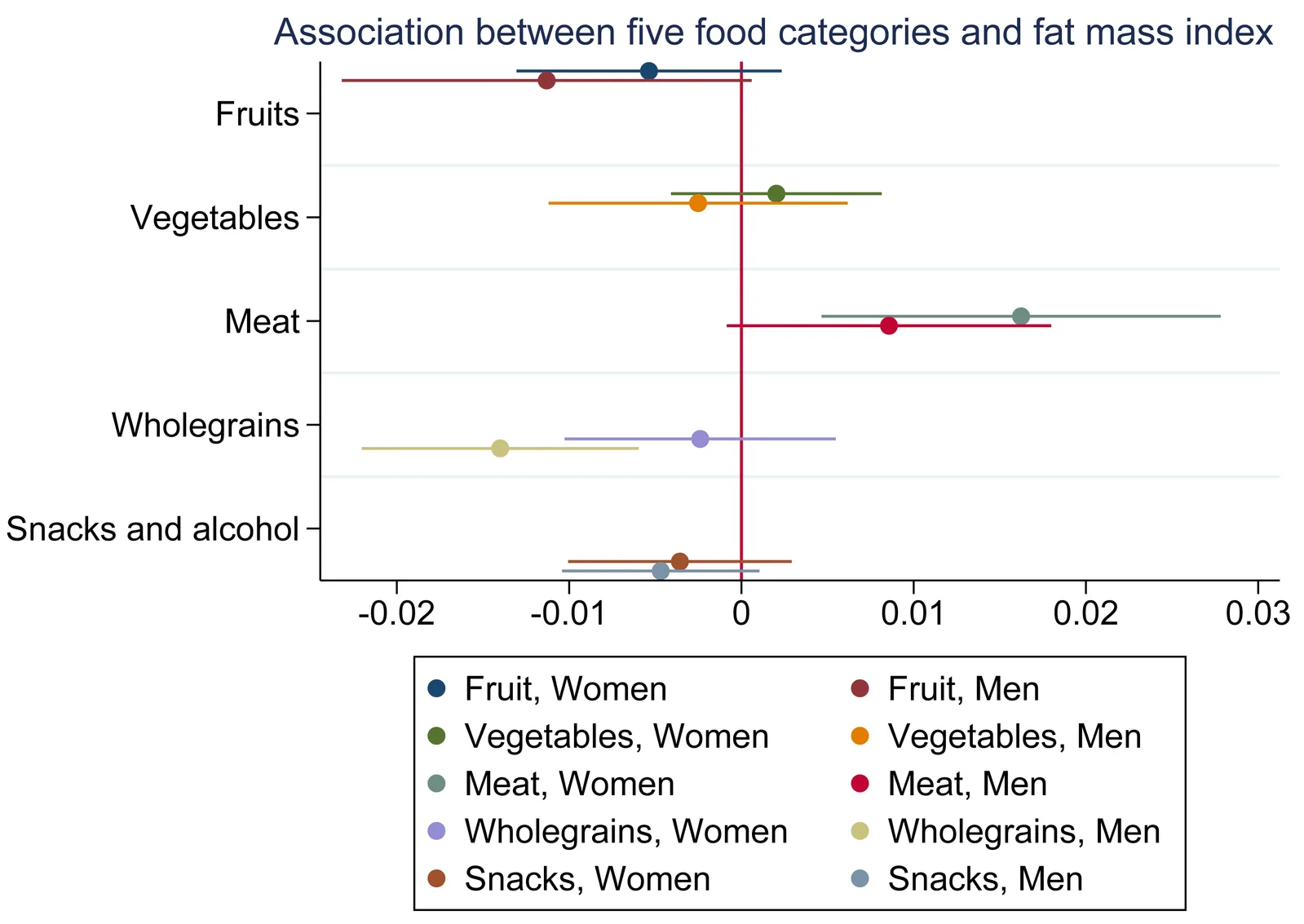
Plot of coefficients and 95% confidence intervals from five multivariable linear regression analyses with outcome FMI, one for each type of food, and by sex. Only. Only the results for the dietary components (in number of consumptions per week) are shown. All analyses were conducted with the same covariables as in the models in Figs. 1 and 2. A coefficient of 0.016, as seen *e.g.* for number of meat consumptions per week in women, implies an increase in FMI by 0.016 units per consumption on the square root scale.

*VAT:* In women, the consumption of fruits was negatively associated with VAT (*p* = 0.01). In men, the consumption of fruits (*p* = 0.035), and wholegrain foods (*p* = 0.001) was negatively associated with VAT. For meat, as well as for sweet and salty snacks, there was no association with VAT in neither sex in our sample (all *p* > 0.05). For all results see Fig. 5.

**Figure 5:**
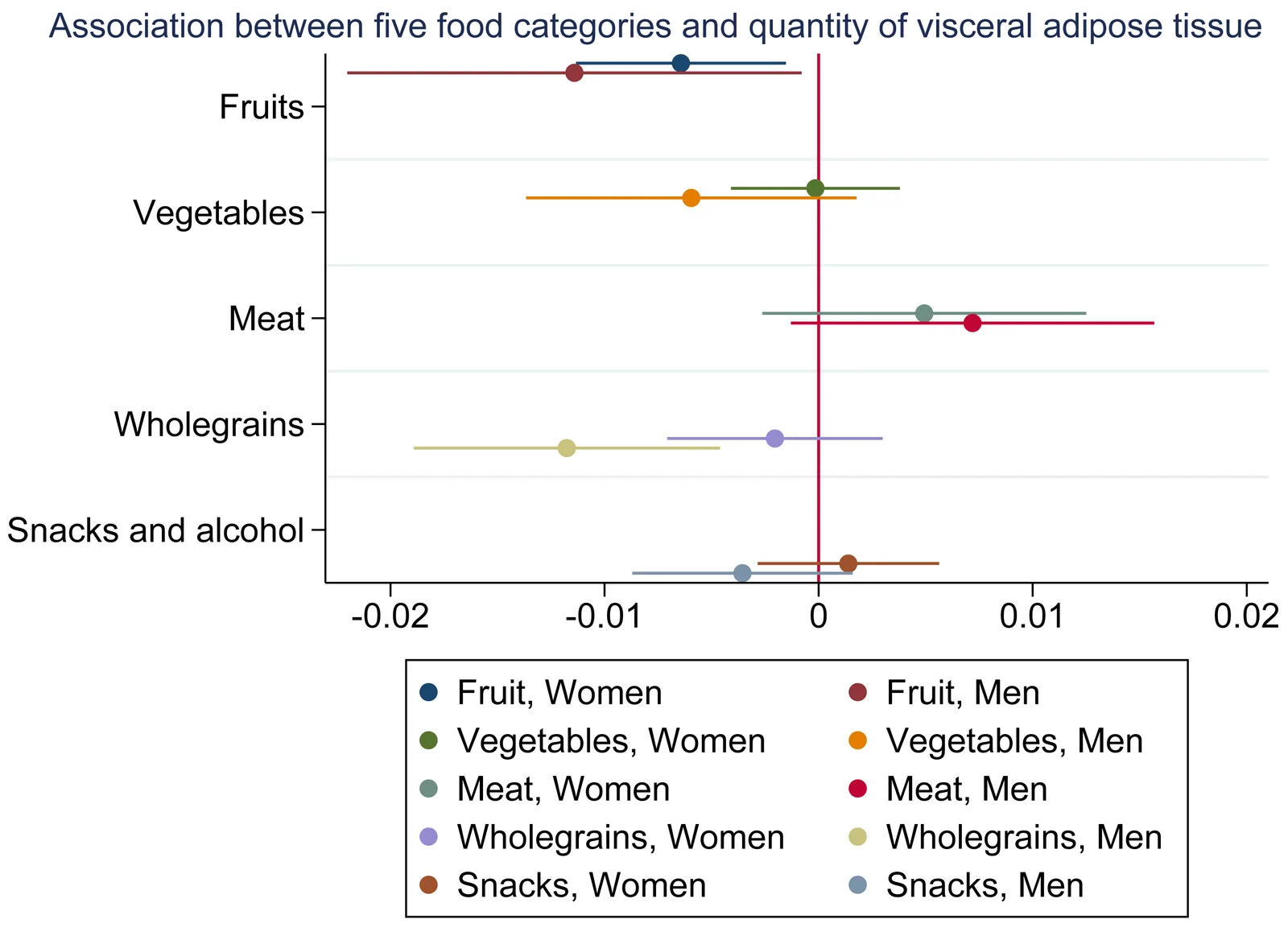
Plot of coefficients and 95% confidence intervals from five multivariable linear regression analyses with outcome VAT, one for each type of food, and by sex. Only the results for the dietary components (in number of consumptions per week) are shown. All analyses were conducted with the same covariables as in the models in Figs. 1 and 2. A coefficient of 0.005, as seen *e.g.*, for number of meat consumptions per week in women, implies an increase in VAT by 0.005 units per consumption on the square root scale.

## Discussion

Our study analysed the association of the modifiable lifestyle factors diet and PA, with BMI and body composition, corrected for education, place of residence, age, and sex. For both sexes, age was the strongest predictor of adiposity, showing a positive association with FMI and VAT, while PA was negatively associated with FMI and VAT.

A special emphasis was given to sex differences in the associations between lifestyle factors and body composition outcomes, as our analyses showed a positive interaction with sex for the associations between SMI and diet/PA, as well as for VAT and diet/PA. Other studies confirm the sex-specific impact of diet healthiness on several health outcomes (Sulis et al., 2025). In our study, overall healthiness of diet was more strongly associated with BMI and SMI in women, while specific food components were associated with VAT in men. In fact, regarding dietary components, consuming fruits and whole grains was associated with lower FMI and VAT in men, while in women, we found this association with VAT only with consuming fruits. Our results align with findings in the literature. Various studies reported health benefits including a negative association with BMI by consuming fruits and vegetables, reporting a nonlinear increase of benefits with increasing consumption (Schlesinger et al., 2019; Seifu et al., 2021). Accordingly, the Swiss dietary recommendations include the intake of 5 servings per day (2 fruits, 3 vegetables). However, our cross-sectional observational study does not allow for causal inferences. It could be that women with low VAT consume more fruits, and not that fruits lead to a lower VAT in women. This caveat of reverse causality is valid for all our results.

Regarding whole grain consumption, we found a negative association with FMI and VAT, especially in men. The current published data are heterogenous in case of whole grain intake. A recently published review found a negative association with BMI and VAT (Wang et al., 2020), while others did not (Sadeghi et al., 2020). A meta-analysis of observational studies and randomized controlled trials (RCT) found an association in the cross-sectional studies, but not in RCTs (Maki et al., 2019). As a recent systematic review found, different methods of calculating and reporting grain intake, as well as the definition of whole grain product, affect the results on possible influences on body weight (Kissock, Neale & Beck, 2021). The conclusion was that studies calculating whole grain intake in total grams, or studies reporting food items with ≥25% whole grain in combination with other specific foods, show a significant negative effect on BMI (Kissock, Neale & Beck, 2021). Due to these studies the Swiss dietary recommendation suggests eating preferably whole grains when consuming grains (Marques-Vidal, 2022).

Regarding meat, studies have shown an association between consuming red meat and risk of CVD and T2D (Van Eekelen et al., 2019; Schwingshackl et al., 2017). However, only few prospective studies are available to evaluate the association of meat consumption with obesity (Schlesinger et al., 2019). In our study, in women a high consumption of meat was associated with increased SMI and FMI. As women eat less meat than men overall, it could be that this result reflects a relative lack of proteins in women, resulting in a positive association of body composition measures with increasing meat consumption. In fact, we found an increase in SMI by 0.007 units per consumption of meat on the square root scale in women, compared to a woman of the same height that eats no meat. This increase due to nutrition alone (without taking physical activity into account) is in line with previous studies and represents a significant difference at the population level (Tagawa et al., 2020; Silva et al., 2021). However, the above caveat about causality holds true again. Therefore, it could also be that women with a higher SMI prefer to eat more meat.

Interestingly, in men a higher meat consumption was not associated with higher FMI or VAT. Since there are causal mechanisms that suggest a link between processed meat consumption and the risk of CVD, T2D and certain cancers (Rohrmann & Linseisen, 2016), there should be more studies on this subject in the future. A further interesting development is the increase in plant based and other alternative protein sources such as insects, bio-engineered meat, and other novel food items derived from cellular agriculture. Few studies assessed the health impact of these food items, but it seems that there might be some health benefit (Selinger et al., 2023; Quintieri et al., 2023). With increasing popularity, these alternative protein sources should be targeted more in future studies on health impact.

Interestingly, the food components considered as less healthy such as sweet and salty snacks were not associated with a higher bodyfat in either sex in our study. An association between sweet snacks and increased hepatic triglyceride content was shown in a recent study (Van Eekelen et al., 2019). An association with BMI and FMI is probable but could not be shown in the same study. Several studies investigated the association between ultra-processed food (UPF), that also contains sweet and salty snacks, and obesity, and were evaluated in a meta-analysis. The meta-analysis showed a clearly positive association between UPF and obesity as well as VAT (Lane et al., 2021). The cross-sectional Swiss National Nutrition Survey showed that especially women in the highest quintile of UPF consumption showed a proportionally higher risk of obesity, VAT and higher BMI-waist circumference composite outcome. This was not observed in men with similar intake of UPF (Pestoni et al., 2021).

Overall, our results are similar to the recent scientific report of the Swiss dietary recommendations in 2022. We observed an association between adherence to these recommendations and lower FMI and BMI, especially in women (Marques-Vidal, 2022). Similar results are seen in recently published studies that show an association between adherence to official dietary recommendations, which are similar to the Swiss recommendations, and reduced risk for CVD, T2D and overall mortality (Panizza et al., 2020; Schwingshackl et al., 2017). However, overall, our study population did not follow thoroughly the official dietary recommendations, with a better result for women than men. In both sexes together, only 34% of our study population followed at least four of five recommendations. In a representative Swiss dietary survey carried out in 2014–2015, up to 40% of participants followed three or more official recommendations (Chatelan et al., 2017). Our results are therefore comparable to the Swiss dietary survey.

A further interesting finding in our study was the low impact of PA on BMI in women. While in men, PA was clearly negatively associated with BMI, we did not find an effect in women. A reason could be the simultaneous increase in SMI and decrease in FMI with increasing PA in women. In this context, BMI shows some weaknesses in measuring physical health, as it cannot distinguish between fat and muscular mass (Staub et al., 2018). In fact, people with high VAT but low muscular mass can have a normal BMI while suffering from central obesity (Mili et al., 2021; Chua et al., 2021).

Overall, we observed a sex difference in accumulation of fat tissue, and a lower association of specific food components and PA with body composition outcomes in women compared to men. Several hypotheses addressing this topic were published, including evolutionary explanations. Our findings are in line with evolutionary explanations, such as fat tissue as an energy storage for surviving food shortages in the past, especially in women (Wells, 2012; Pond, 1999). During pregnancy and breastfeeding, the female fat storage is regarded as crucial to ensure reproduction even through periods of food shortage (Vernon & Pond, 1997). Fat tissue is also postulated to be necessary during pregnancy and lactation for brain development in babies (Crawford, 2012). Similarly, the stronger positive association between protein intake and increase in FMI and SMI in women could be interpreted as an adaptation to the specific physiological needs during pregnancy and lactation. However, such adaptations might turn into risk factors for chronic diseases in modern environments. In fact, continuous availability of food and lack of food shortage in modern time is regarded as leading to an evolutionary mismatch, resulting in growing obesity rates in developed and developing countries as postulated by the thrifty genotype hypothesis (Neel, 1962). For women with their postulated adaptations to pregnancy and lactation it is even more difficult to avoid obesity, as the human genetics still has not adapted to modern living conditions (Speakman, 2008). A similar evolutionary mismatch might apply to generally low levels of PA in modern societies. In this context, the value of recreational PA becomes evident as an important factor to improve body composition measures. Moreover, many people nowadays experience a *developmental mismatch*—either through rapid economic development and the industrialization of food production in their own country, or through migration to another country—experiencing malnutrition in early life, which leads to a thrifty phenotype, but overnutrition later in life, which leads to obesity (Gluckman, Hanson & Low, 2019).

A strength of our study was that body weight was measured and not collected *via* questionnaire. This led to a higher precision and eliminated potential reporting bias. In addition to BMI, we measured body composition, which allowed us to better understand the associations between lifestyle factors and different types of fat mass, and muscle mass. Our methods allowed for a fast, cost-effective, and precise, noninvasive collection of data without any side effects for probands. Furthermore, our study sample is heterogenous in terms of age and sex.

A limitation of our study was the lack of heterogeneity regarding ethnicity, education, and socio-economic status. The participants were mainly recruited *via* invitations, mailing lists, and advertisements at universities. For that reason, our sample consisted of rather urban, well-educated people (tertiary education of 75%, compared to the Swiss general population with a tertiary education of 45% in 2021) (BFS, 2023). Our study is therefore not representative for the general population of Switzerland. Additionally, as this was an observational study, we cannot infer causality between the variables. As discussed, it could be that women with low VAT consume more fruits, and women with a higher SMI consume more meat, without proving a direct effect of food on body composition. Furthermore, even though we accounted for several known potential confounders in our analyses, we cannot exclude the existence of further confounders that we did not assess.

Furthermore, the timing of BIA measurements varied during the day, and even though we instructed the participants not to eat, drink, and exercise before the measurement, and we corrected for the last time of drinking, residual confounding cannot be excluded. In fact, even though we corrected our analyses for a multitude of variables, potential remaining confounding by unmeasured variables cannot be excluded in general. For instance, we did not assess total energy intake with our questionnaire. The fact that we could not correct the associations between diet and body composition for total energy intake is a limitation that needs to be considered when interpreting the results. Furthermore, even if instructing participants not to drink before the BIA measurements, and even if correcting for the last time of drinking in the analyses, a residual measurement error cannot be excluded.

Finally, we ran several models for the same outcome variable without correcting for multiple testing. This fact must be considered when interpreting the results, especially for weak associations.

### Conclusion

Our study shows that a healthy diet was associated with lower BMI, FMI and VAT. The results are similar to the Swiss dietary recommendations 2022, where a similar association was reported between BMI and food quality. However, the Swiss dietary recommendations did not differentiate between the sexes. In our study we found sex differences in fat accumulation and BMI according to different food consumptions, which could reflect evolutionary adaptations to pregnancy and lactation in women. Furthermore, age seems to be of major importance. Future studies should routinely address sex and age differences, and also investigate causality, *e.g.*, through longitudinal or experimental designs. If confirmed, our results point to the need to adapt prevention programs and dietary recommendations according to population age groups and sex. Specifically, preventive efforts should target obesity and abdominal obesity in association with food quality and PA in a more subgroup and sex specific way.

## Supplemental Information

10.7717/peerj.21821/supp-1 Supplemental Information 1 STROBE checklist

10.7717/peerj.21821/supp-2 Supplemental Information 2 Variable dataset

10.7717/peerj.21821/supp-3 Supplemental Information 3 Results from multivariable linear regression analyses

## Additional Information and Declarations

### Data Availability

The following information was supplied regarding data availability: The data is available in the Supplemental Files.
