# Supplementary material for: Different body components associate differently with diet quality and physical activity in men and in women: a cross-sectional analysis
Source: PeerJ. 2026 Oct 21;14:e21821. doi: 10.7717/peerj.21821 (PMC13695422; doi:10.7717/peerj.21821)
Supplement: Supplementary file 2 [file peerj-14-21821-s003.docx]

**Supplement 1: Results from multivariable linear regression analyses**

| **Association between risk factors and body mass index (BMI) in women** | | | | |  |  |
| --- | --- | --- | --- | --- | --- | --- |
|  |  |  |  |  |  |  |
| Source | SS | df | MS | | Number of obs = | 235 |
|  |  |  |  | | F(16, 218) = | 1.47 |
| Model | 0.36848816 | 16 | 0.02303051 | | Prob > F = | 0.1133 |
| Residual | 3.41946866 | 218 | 0.015685636 | | R-squared = | 0.0973 |
|  |  |  |  | | Adj R-squared = | 0.031 |
| Total | 3.78795681 | 234 | 0.01618785 | | Root MSE = | 0.12524 |
|  |  |  |  |  |  |  |
| **BMI** | **Coefficient** | **SE** | **t** | **p** | **95% LCI** | **95% UCI** |
|  |  |  |  |  |  |  |
| HDI |  |  |  |  |  |  |
| mediocre | -0.0235702 | 0.0209181 | -1.13 | 0.261 | -0.0647978 | 0.0176575 |
| healthy | -0.0577262 | 0.025032 | -2.31 | **0.022** | -0.1070619 | -0.0083904 |
|  |  |  |  |  |  |  |
| PA rec |  |  |  |  |  |  |
| medium | -0.0154371 | 0.0304643 | -0.51 | 0.613 | -0.0754794 | 0.0446052 |
| heavy | -0.0326485 | 0.0300255 | -1.09 | 0.278 | -0.0918259 | 0.0265288 |
|  |  |  |  |  |  |  |
| PA work |  |  |  |  |  |  |
| medium | -0.0022442 | 0.0188282 | -0.12 | 0.905 | -0.0393528 | 0.0348645 |
| heavy | -0.0558916 | 0.0327513 | -1.71 | 0.089 | -0.1204413 | 0.0086581 |
|  |  |  |  |  |  |  |
| Age quartile |  |  |  |  |  |  |
| 2nd | 0.0203747 | 0.0232075 | 0.88 | 0.381 | -0.025365 | 0.0661144 |
| 3rd | 0.0454453 | 0.0233011 | 1.95 | 0.052 | -0.0004789 | 0.0913694 |
| oldest | 0.0877643 | 0.0284956 | 3.08 | **0.002** | 0.0316022 | 0.1439265 |
|  |  |  |  |  |  |  |
| Education |  |  |  |  |  |  |
| higher | -0.0016368 | 0.0348864 | -0.05 | 0.963 | -0.0703946 | 0.0671209 |
| tertiary | -0.0096333 | 0.0345905 | -0.28 | 0.781 | -0.0778078 | 0.0585412 |
|  |  |  |  |  |  |  |
| Residence |  |  |  |  |  |  |
| urban | 0.0173892 | 0.0191791 | 0.91 | 0.366 | -0.0204109 | 0.0551893 |
| rural | 0.0452804 | 0.0320759 | 1.41 | 0.159 | -0.0179382 | 0.108499 |
|  |  |  |  |  |  |  |
| Timepoint drinking | |  |  |  |  |  |
| 1h | -0.0174217 | 0.0200253 | -0.87 | 0.385 | -0.0568897 | 0.0220463 |
| 2h | -0.0574247 | 0.0242816 | -2.36 | **0.019** | -0.1052815 | -0.0095678 |
| >3h | -0.0278471 | 0.0322176 | -0.86 | 0.388 | -0.0913449 | 0.0356507 |
|  |  |  |  |  |  |  |
| Constant | 3.116544 | 0.0496062 | 62.83 | <0.001 | 3.018775 | 3.214313 |

HDI: healthy diet index; PA rec: physical activity during recreation; PA work: physical activity during work; p-values in bold are statistically significant. The reference categories are omitted.

**Association between risk factors and body mass index (BMI) in men**

| Source SS | df | MS |  | | Number of obs = | 183 |
| --- | --- | --- | --- | --- | --- | --- |
|  |  |  |  | | F(16, 166) = | 1.9 |
| Model | 0.46670202 | 16 | 0.029168876 | | Prob > F = | 0.0231 |
| Residual | 2.5425368 | 166 | 0.015316487 | | R-squared = | 0.1551 |
|  |  |  |  | | Adj R-squared = | 0.0737 |
| Total | 3.00923882 | 182 | 0.016534279 | | Root MSE = | 0.12376 |
|  |  |  |  |  |  |  |
| **BMI** | **Coefficient** | **SE** | **t** | **p** | **95% LCI** | **95% UCI** |
|  |  |  |  |  |  |  |
| HDI |  |  |  |  |  |  |
| mediocre | -0.0110935 | 0.0205806 | -0.54 | 0.591 | -0.0517269 | 0.0295399 |
| healthy | 0.0005938 | 0.0374189 | 0.02 | 0.987 | -0.0732844 | 0.074472 |
|  |  |  |  |  |  |  |
| PA rec |  |  |  |  |  |  |
| medium | -0.1008264 | 0.0417657 | -2.41 | **0.017** | -0.1832869 | -0.0183659 |
| heavy | -0.1122154 | 0.0393277 | -2.85 | **0.005** | -0.1898623 | -0.0345686 |
|  |  |  |  |  |  |  |
| PA work |  |  |  |  |  |  |
| medium | 0.0111252 | 0.0233185 | 0.48 | 0.634 | -0.0349139 | 0.0571643 |
| heavy | 0.0220074 | 0.0320571 | 0.69 | 0.493 | -0.0412847 | 0.0852995 |
|  |  |  |  |  |  |  |
| Age quartile |  |  |  |  |  |  |
| 2nd | 0.0199223 | 0.0309946 | 0.64 | 0.521 | -0.0412722 | 0.0811167 |
| 3rd | 0.0313234 | 0.030068 | 1.04 | 0.299 | -0.0280417 | 0.0906885 |
| oldest | 0.042069 | 0.0277618 | 1.52 | 0.132 | -0.0127429 | 0.0968808 |
|  |  |  |  |  |  |  |
| Education |  |  |  |  |  |  |
| higher | -0.0396712 | 0.035077 | -1.13 | 0.26 | -0.1089258 | 0.0295834 |
| tertiary | -0.0178724 | 0.0355866 | -0.5 | 0.616 | -0.0881331 | 0.0523884 |
|  |  |  |  |  |  |  |
| Residence |  |  |  |  |  |  |
| urban | -0.0554889 | 0.0230873 | -2.4 | **0.017** | -0.1010715 | -0.0099064 |
| rural | -0.007336 | 0.0286526 | -0.26 | 0.798 | -0.0639066 | 0.0492345 |
|  |  |  |  |  |  |  |
| Timepoint drinking | |  |  |  |  |  |
| 1h | 0.0204762 | 0.0246663 | 0.83 | 0.408 | -0.0282239 | 0.0691764 |
| 2h | 0.0136526 | 0.0273432 | 0.5 | 0.618 | -0.0403327 | 0.0676379 |
| >3h | 0.0509875 | 0.02902 | 1.76 | 0.081 | -0.0063084 | 0.1082834 |
|  |  |  |  |  |  |  |
| Constant | 3.300653 | 0.0587658 | 56.17 | <0.001 | 3.184628 | 3.416678 |

HDI: healthy diet index; PA rec: physical activity during recreation; PA work: physical activity during work; p-values in bold are statistically significant. The reference categories are omitted.

**Association between risk factors and visceral adipose tissue (VAT) in women**

| Source | SS | df | MS | | Number of obs = | 235 |
| --- | --- | --- | --- | --- | --- | --- |
|  |  |  |  | | F(16, 218) = | 12.38 |
| Model | 12.8729184 | 16 | 0.804557402 | | Prob > F = | 0 |
| Residual | 14.1657066 | 218 | 0.064980305 | | R-squared = | 0.4761 |
|  |  |  |  | | Adj R-squared = | 0.4376 |
| Total | 27.038625 | 234 | 0.11554968 | | Root MSE = | 0.25491 |
|  |  |  |  |  |  |  |
| **VAT** | **Coefficient** | **SE** | **t** | **p** | **95% LCI** | **95% UCI** |
|  |  |  |  |  |  |  |
| HDI |  |  |  |  |  |  |
| mediocre | -0.0485188 | 0.0425758 | -1.14 | 0.256 | -0.132432 | 0.0353941 |
| healthy | -0.091412 | 0.050949 | -1.79 | 0.074 | -0.191828 | 0.0090036 |
|  |  |  |  |  |  |  |
| PA rec |  |  |  |  |  |  |
| medium | -0.1440262 | 0.0620057 | -2.32 | **0.021** | -0.266234 | -0.0218188 |
| heavy | -0.2840727 | 0.0611124 | -4.65 | **<0.001** | -0.40452 | -0.1636259 |
|  |  |  |  |  |  |  |
| PA work |  |  |  |  |  |  |
| medium | -0.000803 | 0.0383221 | -0.02 | 0.983 | -0.076332 | 0.0747261 |
| heavy | 0.0071099 | 0.0666604 | 0.11 | 0.915 | -0.124272 | 0.1384913 |
|  |  |  |  |  |  |  |
| Age quartile |  |  |  |  |  |  |
| 2nd | 0.0793853 | 0.0472354 | 1.68 | 0.094 | -0.013711 | 0.1724818 |
| 3rd | 0.2608455 | 0.0474259 | 5.5 | **<0.001** | 0.1673735 | 0.3543174 |
| oldest | 0.562994 | 0.0579986 | 9.71 | **<0.001** | 0.4486842 | 0.6773039 |
|  |  |  |  |  |  |  |
| Education |  |  |  |  |  |  |
| higher | -0.0437751 | 0.0710061 | -0.62 | 0.538 | -0.183721 | 0.0961712 |
| tertiary | -0.0307787 | 0.0704038 | -0.44 | 0.662 | -0.169538 | 0.1079805 |
|  |  |  |  |  |  |  |
| Residence | |  |  |  |  |  |
| urban | -0.0369462 | 0.0390362 | -0.95 | 0.345 | -0.113883 | 0.0399904 |
| rural | 0.0975751 | 0.0652858 | 1.49 | 0.136 | -0.031097 | 0.2262472 |
|  |  |  |  |  |  |  |
| Timepoint drinking |  |  |  |  |  |  |
| 1h | -0.0174254 | 0.0407586 | -0.43 | 0.669 | -0.097757 | 0.062906 |
| 2h | -0.0400418 | 0.0494217 | -0.81 | 0.419 | -0.137447 | 0.0573638 |
| >3h | -0.0740719 | 0.0655741 | -1.13 | 0.26 | -0.203312 | 0.0551685 |
|  |  |  |  |  |  |  |
| Constant | 0.7463852 | 0.1009662 | 7.39 | <0.001 | 0.5473903 | 0.9453801 |

HDI: healthy diet index; PA rec: physical activity during recreation; PA work: physical activity during work; p-values in bold are statistically significant. The reference categories are omitted.

**Association between risk factors and visceral adipose tissue (VAT) in men**

| Source | SS | df | MS | Number of obs = | | | 183 |
| --- | --- | --- | --- | --- | --- | --- | --- |
|  |  |  |  | F(16, 166) = | | | 11.91 |
| Model | 28.7056778 | 16 | 1.79410486 | Prob > F = | | | 0 |
| Residual | 24.997031 | 166 | 0.150584524 | R-squared = | | | 0.5345 |
|  |  |  |  | Adj R-squared = | | | 0.4897 |
| Total | 53.7027088 | 182 | 0.295069828 | Root MSE = | | | 0.38805 |
|  |  |  |  | |  |  |  |
| **VAT** | **Coefficient** | **SE** | **t** | | **p** | **95% LCI** | **95% UCI** |
|  |  |  |  | |  |  |  |
| HDI |  |  |  | |  |  |  |
| mediocre | -0.0423204 | 0.064531 | -0.66 | | 0.513 | -0.1697276 | 0.0850868 |
| healthy | -0.1778435 | 0.1173279 | -1.52 | | 0.131 | -0.4094907 | 0.0538037 |
|  |  |  |  | |  |  |  |
| PA rec |  |  |  | |  |  |  |
| medium | -0.3952525 | 0.1309576 | -3.02 | | **0.003** | -0.6538097 | -0.1366954 |
| heavy | -0.5576033 | 0.1233129 | -4.52 | | **<0.001** | -0.8010671 | -0.3141394 |
|  |  |  |  | |  |  |  |
| PA work |  |  |  | |  |  |  |
| medium | 0.0658597 | 0.0731159 | 0.9 | | 0.369 | -0.0784972 | 0.2102166 |
| heavy | 0.0888904 | 0.1005158 | 0.88 | | 0.378 | -0.1095637 | 0.2873444 |
|  |  |  |  | |  |  |  |
| Age quartile |  |  |  | |  |  |  |
| 2nd | 0.2202626 | 0.0971844 | 2.27 | | **0.025** | 0.0283859 | 0.4121394 |
| 3rd | 0.3634553 | 0.0942792 | 3.86 | | **<0.001** | 0.1773145 | 0.5495961 |
| oldest | 0.753628 | 0.087048 | 8.66 | | **<0.001** | 0.581764 | 0.9254919 |
|  |  |  |  | |  |  |  |
| Education |  |  |  | |  |  |  |
| higher | 0.048017 | 0.109985 | 0.44 | | 0.663 | -0.1691326 | 0.2651667 |
| tertiary | -0.0020551 | 0.1115829 | -0.02 | | 0.985 | -0.2223596 | 0.2182494 |
|  |  |  |  | |  |  |  |
| Residence | |  |  | |  |  |  |
| urban | -0.1304938 | 0.0723907 | -1.8 | | 0.073 | -0.273419 | 0.0124315 |
| rural | -0.0828319 | 0.0898411 | -0.92 | | 0.358 | -0.2602104 | 0.0945465 |
|  |  |  |  | |  |  |  |
| Timepoint drinking |  |  |  | |  |  |  |
| 1h | 0.012533 | 0.0773419 | 0.16 | | 0.871 | -0.1401676 | 0.1652336 |
| 2h | 0.1759733 | 0.0857355 | 2.05 | | **0.042** | 0.0067008 | 0.3452457 |
| >3h | 0.2032046 | 0.090993 | 2.23 | | **0.027** | 0.0235518 | 0.3828574 |
|  |  |  |  | |  |  |  |
| Constant | 1.156829 | 0.1842619 | 6.28 | | <0.001 | 0.7930303 | 1.520628 |

HDI: healthy diet index; PA rec: physical activity during recreation; PA work: physical activity during work; p-values in bold are statistically significant. The reference categories are omitted.

**Association between diet components and fat mass index (FMI) in women**

| **FMI** | **Coefficient** | **SE** | **t** | **p** | **95% LCI** | **95% UPC** |
| --- | --- | --- | --- | --- | --- | --- |
|  |  |  |  |  |  |  |
| Fruits | -0.0053615 | 0.0039037 | -1.37 | 0.171 | -0.0130555 | 0.0023324 |
| Vegetables | 0.0020309 | 0.0031034 | 0.65 | 0.514 | -0.0040858 | 0.0081476 |
| Meat | 0.0162348 | 0.0058756 | 2.76 | **0.006** | 0.0046533 | 0.0278163 |
| Wholegrains | -0.0023888 | 0.0039908 | -0.6 | 0.55 | -0.0102552 | 0.0054776 |
| Snacks and alcohol | -0.00357 | 0.0032913 | -1.08 | 0.279 | -0.0100576 | 0.0029176 |

p-values in bold are statistically significant. Only the main outcome per regression is shown.

**Association between diet components and fat mass index (FMI) in men**

| **FMI** | **Coefficient** | **SE** | **t** | **p** | **95% LCI** | **95% UPC** |
| --- | --- | --- | --- | --- | --- | --- |
|  |  |  |  |  |  |  |
| Fruits | -0.0113009 | 0.0060257 | -1.88 | 0.062 | -0.0231977 | 0.0005959 |
| Vegetables | -0.0025111 | 0.004396 | -0.57 | 0.569 | -0.0111904 | 0.0061683 |
| Meat | 0.0085672 | 0.0047696 | 1.8 | 0.074 | -0.0008501 | 0.0179846 |
| Wholegrains | -0.0140005 | 0.0040699 | -3.44 | **0.001** | -0.0220373 | -0.0059637 |
| Snacks and alcohol | -0.0046847 | 0.0029016 | -1.61 | 0.108 | -0.0104138 | 0.0010444 |

p-values in bold are statistically significant. Only the main outcome per regression is shown.

**Association between diet components and skeletal muscle mass index (SMI) in women**

| **FMI** | **Coefficient** | **SE** | **t** | **p** | **95% LCI** | **95% UPC** |
| --- | --- | --- | --- | --- | --- | --- |
|  |  |  |  |  |  |  |
| Fruits | -0.0003463 | 0.0012904 | -0.27 | 0.789 | -0.0028897 | 0.002197 |
| Vegetables | 0.0003808 | 0.0010223 | 0.37 | 0.71 | -0.0016341 | 0.0023958 |
| Meat | 0.0073216 | 0.0018921 | 3.87 | **<0.00** | 0.003592 | 0.0110513 |
| Wholegrains | -0.001282 | 0.0013104 | -0.98 | 0.329 | -0.0038649 | 0.001301 |
| Snacks and alcohol | -0.0013835 | 0.0011004 | -1.26 | 0.21 | -0.0035524 | 0.0007855 |

**Association between diet components and skeletal muscle mass index (SMI) in men**

| **FMI** | **Coefficient** | **SE** | **t** | **p** | **95% LCI** | **95% UPC** |
| --- | --- | --- | --- | --- | --- | --- |
|  |  |  |  |  |  |  |
| Fruits | -0.0013671 | 0.0022042 | -0.62 | 0.536 | -0.0057189 | 0.0029847 |
| Vegetables | 0.0002679 | 0.0015946 | 0.17 | 0.867 | -0.0028803 | 0.0034162 |
| Meat | 0.0034074 | 0.0017254 | 1.97 | 0.05 | 7.23E-07 | 0.0068141 |
| Wholegrains | -0.0022788 | 0.0014925 | -1.53 | 0.129 | -0.0052261 | 0.0006684 |
| Snacks and alcohol | -0.0012151 | 0.0010565 | -1.15 | 0.252 | -0.003301 | 0.0008709 |

**Association between diet components and visceral adipose tissue (VAT) in women**

| **FMI** | **Coefficient** | **SE** | **t** | **p** | **95% LCI** | **95% UPC** |
| --- | --- | --- | --- | --- | --- | --- |
|  |  |  |  |  |  |  |
| Fruits | -0.0064321 | 0.0024879 | -2.59 | **0.01** | -0.0113355 | -0.0015286 |
| Vegetables | -0.0001507 | 0.0020014 | -0.08 | 0.94 | -0.0040954 | 0.0037939 |
| Meat | 0.0049356 | 0.0038381 | 1.29 | 0.2 | -0.0026297 | 0.0125009 |
| Wholegrains | -0.0020402 | 0.0025527 | -0.8 | 0.425 | -0.0070719 | 0.0029915 |
| Snacks and alcohol | 0.0013884 | 0.0021498 | 0.65 | 0.519 | -0.0028491 | 0.0056259 |

**Association between diet components and visceral adipose tissue (VAT) in men**

| **FMI** | **Coefficient** | **SE** | **t** | **p** | **95% LCI** | **95% UPC** |
| --- | --- | --- | --- | --- | --- | --- |
|  |  |  |  |  |  |  |
| Fruits | -0.0114072 | 0.0053755 | -2.12 | **0.035** | -0.0220203 | -0.000794 |
| Vegetables | -0.005947 | 0.0039098 | -1.52 | 0.13 | -0.0136663 | 0.0017724 |
| Meat | 0.0071924 | 0.0042985 | 1.67 | 0.096 | -1.29E-03 | 0.0156795 |
| Wholegrains | -0.0117641 | 0.0036208 | -3.25 | **0.001** | -0.0189142 | -0.004614 |
| Snacks and alcohol | -0.0035579 | 0.0026056 | -1.37 | 0.174 | -0.0087025 | 0.0015868 |

**Codebook raw data**

Last time of drinking

1) less than one hour ago
2) one hour ago
3) two hours ago
4) three or more hours ago

Place of residence

1) urban
2) suburban
3) rural

Sex

0) female
1) male

Age

1) youngest quartile
2) second quartile
3) third quartile
4) oldest quartile

Healthy eating index

1) unhealthy
2) mediocre
3) healthy

Physical activity leisure

1) light
2) medium
3) heavy

Physical activity work

1) light
2) medium
3) heavy

Education

1) basic education
2) higher education
3) tertiary education
